# Disease-related compound identification based on deeping learning method

**DOI:** 10.1038/s41598-022-24385-1

**Published:** 2022-11-29

**Authors:** Bin Yang, Wenzheng Bao, Jinglong Wang, Baitong Chen, Naoki Iwamori, Jiazi Chen, Yuehui Chen

**Affiliations:** 1 School of Information Science and Engineering, Zaozhuang University, Zaozhuang, 277160 China; 2 School of Information and Electrical Engineering, Xuzhou University of Technology, Xuzhou, 221018 China; 3 College of Food Science and Pharmaceutical Engineering, Zaozhuang University, Zaozhuang, 277160 China; 4 Xuzhou First People’s Hospital, Xuzhou, 221000 China; 5 Laboratory of Zoology, Graduate School of Bioresource and Bioenvironmental Sciences, Kyushu University, Fukuoka-shi, Fukuoka Japan; 6 School of Information Science and Engineering, University of Jinan, Jinan, China

**Keywords:** Computational biology and bioinformatics, Drug discovery

## Abstract

Acute lung injury (ALI) is a serious respiratory disease, which can lead to acute respiratory failure or death. It is closely related to the pathogenesis of New Coronavirus pneumonia (COVID-19). Many researches showed that traditional Chinese medicine (TCM) had a good effect on its intervention, and network pharmacology could play a very important role. In order to construct "disease-gene-target-drug" interaction network more accurately, deep learning algorithm is utilized in this paper. Two ALI-related target genes (REAL and SATA3) are considered, and the active and inactive compounds of the two corresponding target genes are collected as training data, respectively. Molecular descriptors and molecular fingerprints are utilized to characterize each compound. Forest graph embedded deep feed forward network (forgeNet) is proposed to train. The experimental results show that forgeNet performs better than support vector machines (SVM), random forest (RF), logical regression (LR), Naive Bayes (NB), XGBoost, LightGBM and gcForest. forgeNet could identify 19 compounds in Erhuang decoction (EhD) and Dexamethasone (DXMS) more accurately.

## Introduction

Internal and external etiology can lead to self-stable regulation disorder, which could change a series of metabolisms, functions and structures. Abnormal life activity processes are manifested as abnormal symptoms, signs and behavior^1,2^. Under certain conditions, the abnormal life activity processes caused by the disturbance of homeostasis after the damage of the disease cause the disease^3,4^. Traditional Chinese medicine (TCM) has been utilized to treat diseases for thousands of years^5–7^. Traditional Chinese medicine is a kind of material with the function of rehabilitation and health care, which could be utilized to prevent, treat and diagnose diseases under the guidance of TCM theory^8–11^.

Traditional Chinese medicine mainly comes from natural medicine and its processed products, including plant medicine, animal medicine, mineral medicine and some chemical and biological products^12,13^. The most important feature of traditional Chinese medicine in treating diseases is to pay attention to the adjustment of the functions of viscera and organs, and the balance and coordination between them. The focus of traditional Chinese medicine treatment is not that the human body is infected with the specific bacteria, virus and other pathogenic factors, but the specific reaction of the human body after these pathogenic factors act on the human body^14,15^. The purpose of treatment is to enhance the disease resistance and recovery ability of human body. To kill bacteria and relieve symptoms are mainly achieved by enhancing the body's own functions. In recent years, traditional Chinese medicine has certain advantages in the treatment of pneumonia^16^, shock^17^, convulsion^18^, hemorrhage^19^, acute respiratory failure^20^, renal failure^21^, heart failure^22^, cerebrovascular accident^23^, etc. it is not only effective, but also safe and simple, with few adverse reactions.

In the past decade, with the rapid development of sequencing technology, a large number of genomics data such as genomics, proteomics, metabonomics and so on, have been generated, which has led to the changes in the research of traditional Chinese medicine for diseases. Network pharmacology has been proposed, which was developed on the basis of the rapid development of systems biology and computer technology, generating the "disease-gene-target-drug" interaction network. Through network analysis, we can systematically and comprehensively observe the intervention and influence of drugs on the disease network, reveal the mystery of the synergistic effect of multi branch drugs on the human body, and find out the multi-target new drugs with high efficiency and low toxicity. Network pharmacology of traditional Chinese medicine has become a new idea for drug mechanism research and new drug development^24–28^. Lu et al. utilized network pharmacology and molecular docking technology to study the mechanism of Shaoyao Decoction in the treatment of ulcerative colitis, and found that Shaoyao decoction can improve the pathological damage of colon^29^. Liu et al. collected the main active components of Portulacae Herba, constructed interaction network of target proteins of liver cancer, and found that ketones may be the main material basis of its anti-liver cancer, which is related to the regulation of MAPK signaling pathway^30^. Liu et al. utilized network pharmacology to screen 102 active components of Danzhi Xiaoyao Powder, 147 corresponding targets and 52 intersecting targets with insomnia, and obtained the key components, key targets and key pathways of Danzhi Xiaoyao Powder in the treatment of insomnia^31^. Yang et al. presented network pharmacology to analyze the potential anti-tumor mechanisms of the main active components of Prunella vulgaris systematically at the molecular level^32^. Shen et al. discussed the possible mechanism of Wuling Powder in the treatment of diabetic nephropathy by network pharmacology, and found that Wuling Powder may reduce renal cell damage by regulating apoptosis related proteins, such as Caspases family protein and BCL2 Protein family^33^.

In the recent years, data mining methods have been applied to extract useful information from lots of TCM data^33^. Ren et al. utilized data mining methods to screen out 47 prescriptions, and found out 14 core drugs and 7 new prescriptions in order to search the medication rules and mechanism of TCM in the treatment of carotid atherosclerosis (CAS)^34^. Ga et al. utilized data mining method to select the top five active components of each Tibetan medicine with high frequency and network pharmacology was utilized to analyze the mechanism of Tibetan medicine in the treatment of high altitude polycythemia^35^. In order to study the medication rule of TCM intervention in iron death, Ou et al. constructed target-compound, compound-TCM, target-compound-TCM network, and frequency statistics was utilized to show that bitter and pungent herbs were the main herbs that could interfere with iron death, while cold herbs were the main ones, which mainly belonged to liver and lung meridians^36^. Pan et al. reprocessed a large number of Chinese medicine prescriptions for the treatment of primary liver cancer, and by analysis of data mining and network pharmacology medication regularity of effective traditional Chinese medicine prescriptions in the treatment of primary liver cancer was obtained^37^. Zheng et al. presented four classifiers to infer compound-target interaction network in the process of network pharmacology analysis^38^.

In order to better mine omics data and construct "disease-gene-target-drug" interaction network, deep learning model was utilized in this paper. Taking acute lung injury (ALI) disease as an example, we selected two ALI-related target genes (REAL and SATA3), which have been verified biology experiment. The active compounds are collected from BindingDB database for two key target genes as positive samples. The inactive compounds are generated from DUD-E as the negative samples. The different molecular descriptors and molecular fingerprints are utilized to characterize each compound, which form the full feature set and contain 374 features. With full feature set collected, forest graph embedded deep feed forward network is trained, which is utilized to identify the compounds in Erhuang decoction (EhD) and Dexamethasone (DXMS) for the treatment of acute lung injury.

## Methods

### forgeNet

**Figure 1:**
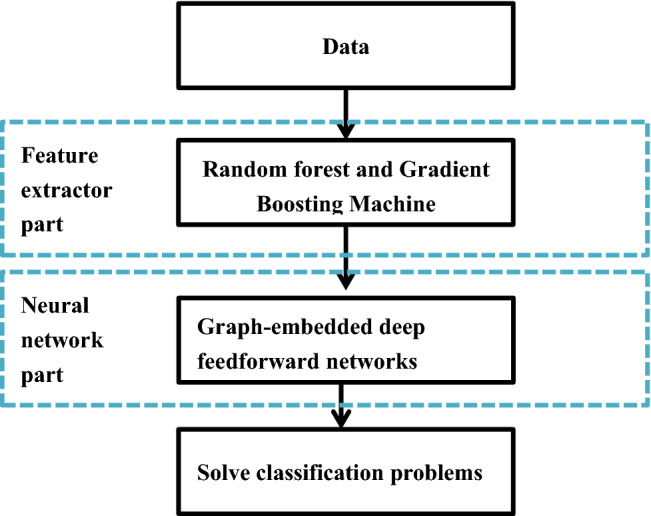
The flowchart of forgeNet algorithm.

Forest graph-embedded deep feedforward network (forgeNet) is a novel machine learning algorithm, which has been successfully applied to solve classification problem with TCGA RNA-seq data. The flowchart of forgeNet is depicted in Fig. 1. From Fig. 1, it could be seen that this method contains two parts: feature graph construction and deep neural network. Compared with deep learning models, forgeNet solves the dimension problem of biological data and is more robust. The algorithm is described as follows^39^.

- Step 1: feature graph construction

**Figure 2:**
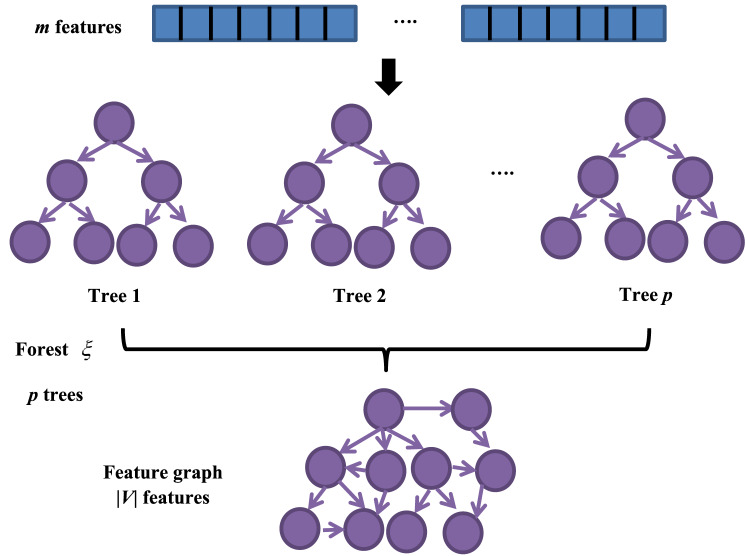
Feature graph construction.

The flowchart of feature graph construction is depicted in Fig. 2. Before the labeled training data are input into classifier, the features of the data need to be extracted. In forgeNet, the used forest $\xi$ contains $p$ decision tree (DT). With the labeled training data, the forest is fitted and $p$ DT are generated ($\xi (\theta ) = \{ T_{1} (\theta_{1} ),\;T_{2} (\theta_{2} ),\; \ldots ,\;T_{p} (\theta_{p} )\}$, $\theta_{i}$ is a parameter). Meanwhile if binary tree is regarded as a special case of directed graph, we can gain the following graph set.

1 $$ \Phi = \{ G_{1} (V_{1} ,\;E_{1} ),\; \ldots ,G_{i} (V_{i} ,\;E_{i} ),\; \ldots ,\;G_{N} (V_{p} ,\;E_{p} )\} . $$

where $V_{i}$ and $E_{i}$ represents vertex set and edge set of $G_{i}$.

To integrate the directed graph set $\Phi$, the final aggregated graph can be gained by the following formula.

2 $$ G(V,E) = \bigcup\limits_{i = 1}^{p} {G_{i} } . $$

- Step 2: deep neural network

The feature graph obtained the previous step are embedded into this part. With the processed features graph-embedded deep feedforward networks (GEDFN) is used to train and make the classification for the unknown data^12^. Every layer of GEDFN is introduced as followed.

3 $$ \begin{gathered} Z_{1} = \sigma (X(W_{in} \Theta G) + b_{in} ), \hfill \\ \ldots \hfill \\ Z_{k + 1} = \sigma (Z_{k} W_{k} + b_{k} ), \hfill \\ \ldots \hfill \\ Z_{out} = \sigma (Z_{l} W_{l} + b_{l} ), \hfill \\ y = soft\max (Z_{out} W_{out} + b_{out} ). \hfill \\ \end{gathered} $$

where $X$ is input data,$Z_{k}$ is the $k - th$ hidden layers, $\Theta$ denotes Hadamard product, $W_{k}$ and $b_{k}$ are the weights and bias of the $k - th$ hidden layer, respectively. $\sigma ( \cdot )$ is an activation function, which could be sigmoid, hyperbolic tangent or rectifiers.

### Inference algorithm

**Figure 3:**
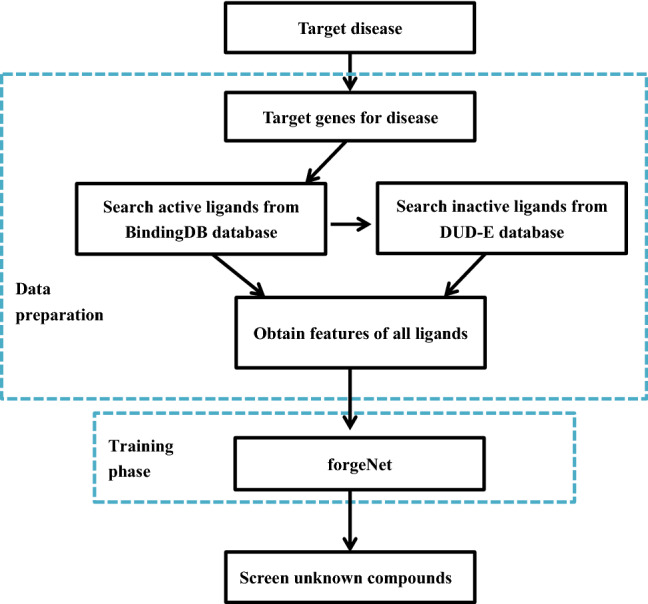
The flowchart of ALI-related compound identification.

In order to construct "disease-gene-target-drug" interaction network more accurately, an ALI-related compound identification based on deep learning model and target genes is proposed. The flowchart is depicted in Fig. 3 and the detailed process is given as follows.

1. Data preparation. Two key target genes: signal transducer and activator of transcription 3 (STAT3), and nuclear transcription factor- κ B/p65 (nuclear factor kappa, B/p65, REAL) were proved to be mainly involved in the key pathways related to acute lung injury (ALI), and losely related to ALI diseases in the literature^40^. Then the BindingDB database (http://www.bindingdb.org/bind/index.jsp) is searched for the known active compounds of these two key target genes^38^. The active ligands are screened with the condition that IC50 < 5000 nmol L^−1^. The collected active compounds are labeled as positive samples. In order to collect the negative samples, 20% of the active ligands are randomly selected and uploaded to DUD-E database (http://dude.docking.org/) to generate the inactive ligands^41^. Active and inactive compound sets form the dataset. The structure of each compound is Simplified Molecular Input Line Entry System (SMILE), so the molecular descriptors and molecular fingerprints of each compound must be obtained as the feature vectors. In this paper, RDKit package is utilized to create the molecular descriptors and molecular fingerprints of each ligand. Molecular descriptors contains 208 features, such as topological polar surface area (TPSA) descriptor, number of valence electros, number of radical electrons, charge information and number of Aliphatic Carbocycles. MACCS fingerprints contains 166 molecular characteristic sites, such as Atom Pairs, topological torsions.
2. Model training. According to the collected data, the feature vector of each ligand is used as input for forgeNet. After training phase, the unknown compounds are screened for the target disease.

## Experiments

In this section, active and inactive ligands of two key target genes: REAL and SATA3 about ALI disease are collected. For REAL, 966 ligands are collected, which contain 146 positive samples and 820 negative samples (Data1). For SATA3, 193 active ligands and 1210 inactive ligands are collected (Data2). Molecular descriptors and molecular fingerprints of each ligand could be obtained, which contains 374 features. In order to better reflect the effectiveness of forgeNet, three classical classifiers (SVM^42^, RF^43^, logical regression (LR), Naive Bayes (NB), XGBoost, LightGBM and gcForest^44^) are utilized to identify the compounds associated with diseases. Five evaluation criteria of classifier performance are utilized, which are *SN*, *SP*, *Kappa*, *MCC* and *F*1, respectively.

### Model test

In order to test the generalization and stability of forgeNet, threefold, fivefold and tenfold cross validation methods are utilized. For each cross validation method, 10-repeat experiments are implemented. Identification averaged performances (Mean ± SD) of eight methods with Data1 and Data2 by threefold cross validation, fivefold and tenfold cross validation methods are listed in Tables 1, 2 and 3, respectively. For Table 1, with Data1 it could be seen that NB algorithm has the best SN performance, which is 0.9111 ± 0.021. In terms of SP, Kappa, MCC and F1, LightGBM performs better than other seven methods and forgeNet has the second better performances. With Data2, NB also obtain the highest SN performance, which shows that this method could identify more true ALI-related compounds than other methods, but NB also obtain the worst SP performance, which reveals that this method identifies most of compounds as related ones. From Tables 2 and 3, we also see that with Data1 and Data2, NB algorithm could obtain the best SN performances by fivefold cross validation and tenfold cross validation methods. forgeNet could obtain the highest SP, which shows that this method could identify more unrelated-disease compounds. Although forgeNet can identify less true related compounds than NB, this method could obtain the higher accuracy according to MCC performances. Kappa performances show that forgeNet can make the prediction results more consistent with the actual classification ones with the unbalanced data. *F*1 performances show that on the whole forgeNet could infer components-disease network more accurately than other seven classifiers. Standard Deviation performances of forgeNet also show that this method could obtain the more stable performances.

**Table 1:** Identification performances of eight methods with Data1 and Data2 by threefold cross validation method.

Datasets	Methods	SN	SP	Kappa	MCC	F1
Data1	RF	0.8789 ± 0.038	0.8886 ± 0.0312	0.6385 ± 0.043	0.6597 ± 0.031	0.7042 ± 0.031
	SVM	0.8541 ± 0.018	0.898 ± 0.022	0.6413 ± 0.042	0.657 ± 0.035	0.7053 ± 0.032
	LR	0.8856 ± 0.018	0.9043 ± 0.012	0.6736 ± 0.025	0.6894 ± 0.022	0.7316 ± 0.012
	NB	**0.9111 ± 0.021**	0.6129 ± 0.014	0.2822 ± 0.008	0.3761 ± 0.01	0.446 ± 0.006
	gcForest	0.9103 ± 0.024	0.9154 ± 0.021	0.7145 ± 0.04	0.7294 ± 0.033	0.7648 ± 0.031
	XGBoost	0.8563 ± 0.034	0.8837 ± 0.03	0.6147 ± 0.06	0.635 ± 0.05	0.6848 ± 0.04
	LightGBM	0.8809 ± 0.037	**0.9388 ± 0.022**	**0.7523 ± 0.036**	**0.7594 ± 0.03**	**0.7936 ± 0.028**
	ForgeNet	0.902 ± 0.021	0.9277 ± 0.019	0.7369 ± 0.027	0.747 ± 0.023	0.7819 ± 0.024
Data2	RF	0.8798 ± 0.025	0.8836 ± 0.023	0.6096 ± 0.039	0.6355 ± 0.03	0.6758 ± 0.03
	SVM	0.775 ± 0.026	0.884 ± 0.021	0.546 ± 0.033	0.563 ± 0.027	0.6211 ± 0.025
	LR	0.819 ± 0.034	0.8689 ± 0.035	0.546 ± 0.049	0.572 ± 0.036	0.6239 ± 0.036
	NB	**0.925 ± 0.015**	0.3688 ± 0.014	0.112 ± 0.006	0.2155 ± 0.01	0.3145 ± 0.004
	gcForest	0.828 ± 0.02	0.897 ± 0.014	0.6058 ± 0.025	0.6224 ± 0.021	0.6703 ± 0.019
	XGBoost	0.8408 ± 0.034	0.8687 ± 0.026	0.5572 ± 0.038	0.5848 ± 0.028	0.6333 ± 0.028
	LightGBM	0.8814 ± 0.024	0.8946 ± 0.022	0.634 ± 0.04	0.6563 ± 0.033	0.6951 ± 0.03
	ForgeNet	0.8723 ± 0.02	**0.9143 ± 0.024**	**0.6597 ± 0.03**	**0.673 ± 0.02**	**0.7142 ± 0.03**

Significant values are in bold.

**Table 2:** Identification performances of eight methods with Data1 and Data2 by fivefold cross validation method.

Datasets	Methods	SN	SP	Kappa	MCC	F1
Data1	RF	0.8733 ± 0.024	0.918 ± 0.016	0.6965 ± 0.025	0.7076 ± 0.02	0.749 ± 0.019
	SVM	0.8596 ± 0.023	0.905 ± 0.02	0.6599 ± 0.03	0.6737 ± 0.028	0.7198 ± 0.026
	LR	0.872 ± 0.019	0.9212 ± 0.021	0.7041 ± 0.04	0.7144 ± 0.034	0.7549 ± 0.032
	NB	**0.9222 ± 0.009**	0.5912 ± 0.008	0.2687 ± 0.004	0.3678 ± 0.004	0.4373 ± 0.003
	gcForest	0.9158 ± 0.019	0.9233 ± 0.012	0.7353 ± 0.03	0.7475 ± 0.03	0.7813 ± 0.02
	XGBoost	0.8669 ± 0.04	0.901 ± 0.032	0.6575 ± 0.048	0.6744 ± 0.037	0.7184 ± 0.036
	LightGBM	0.898 ± 0.03	0.9379 ± 0.02	0.7603 ± 0.04	0.7681 ± 0.03	0.8008 ± 0.03
	ForgeNet	0.8999 ± 0.018	**0.9426 ± 0.014**	**0.7703 ± 0.02**	**0.7769 ± 0.021**	**0.8086 ± 0.02**
Data2	RF	0.8848 ± 0.034	0.9009 ± 0.0241	0.6504 ± 0.039	0.6711 ± 0.029	0.7084 ± 0.03
	SVM	0.7933 ± 0.02	0.8974 ± 0.011	0.5842 ± 0.02	0.5982 ± 0.018	0.6515 ± 0.016
	LR	0.8409 ± 0.021	0.8758 ± 0.028	0.5717 ± 0.048	0.5967 ± 0.039	0.6446 ± 0.037
	NB	**0.9383 ± 0.008**	0.3601 ± 0.009	0.1123 ± 0.005	0.2207 ± 0.007	0.3154 ± 0.004
	gcForest	0.8379 ± 0.03	0.8965 ± 0.03	0.6137 ± 0.05	0.6321 ± 0.04	0.6774 ± 0.04
	XGBoost	0.8647 ± 0.036	0.8803 ± 0.022	0.5934 ± 0.031	0.6194 ± 0.024	0.6626 ± 0.023
	LightGBM	0.8968 ± 0.027	0.9002 ± 0.028	0.6565 ± 0.046	0.6783 ± 0.034	0.7136 ± 0.035
	ForgeNet	0.8956 ± 0.026	**0.9044 ± 0.017**	**0.6633 ± 0.035**	**0.6829 ± 0.03**	**0.7192 ± 0.028**

Significant values are in bold.

**Table 3:** Identification performances of eight methods with Data1 and Data2 by tenfold cross validation method.

Datasets	Methods	SN	SP	Kappa	MCC	F1
Data1	RF	0.8862 ± 0.019	0.9143 ± 0.014	0.6961 ± 0.024	0.7092 ± 0.019	0.7492 ± 0.018
	SVM	0.8615 ± 0.026	0.8904 ± 0.028	0.6314 ± 0.04	0.6499 ± 0.033	0.698 ± 0.032
	LR	0.8658 ± 0.0126	0.9314 ± 0.013	0.7234 ± 0.027	0.7306 ± 0.023	0.7701 ± 0.021
	NB	0.9257 ± 0.008	0.5772 ± 0.003	0.2585 ± 0.004	0.3599 ± 0.006	0.4304 ± 0.003
	gcForest	**0.9302 ± 0.014**	0.9158 ± 0.008	0.7261 ± 0.019	0.7417 ± 0.017	0.7742 ± 0.015
	XGBoost	0.8898 ± 0.017	0.894 ± 0.022	0.6549 ± 0.036	0.6748 ± 0.028	0.7173 ± 0.027
	LightGBM	0.9054 ± 0.013	0.9438 ± 0.014	0.779 ± 0.027	0.7851 ± 0.023	0.8159 ± 0.022
	ForgeNet	0.8951 ± 0.015	**0.95 ± 0.01**	**0.7825 ± 0.014**	**0.7867 ± 0.022**	**0.8181 ± 0.018**
Data2	SVM	0.9082 ± 0.023	0.8903 ± 0.025	0.641 ± 0.04	0.6673 ± 0.03	0.7018 ± 0.033
	RF	0.7923 ± 0.023	0.9096 ± 0.008	0.6097 ± 0.015	0.6202 ± 0.014	0.6714 ± 0.012
	LR	0.8341 ± 0.026	0.8927 ± 0.025	0.6019 ± 0.04	0.6208 ± 0.031	0.6677 ± 0.03
	NB	**0.9497 ± 0.009**	0.3431 ± 0.005	0.1082 ± 0.004	0.2196 ± 0.008	0.3132 ± 0.003
	gcForest	0.843 ± 0.019	0.9057 ± 0.017	0.6348 ± 0.031	0.65 ± 0.025	0.6941 ± 0.024
	XGBoost	0.8698 ± 0.044	0.8875 ± 0.035	0.6144 ± 0.05	0.6384 ± 0.037	0.6793 ± 0.039
	LightGBM	0.8999 ± 0.024	0.9114 ± 0.018	0.6827 ± 0.034	0.7007 ± 0.027	0.7346 ± 0.027
	ForgeNet	0.8817 ± 0.032	**0.9276 ± 0.014**	**0.6979 ± 0.026**	**0.7075 ± 0.02**	**0.745 ± 0.02**

Significant values are in bold.

**Figure 4:**
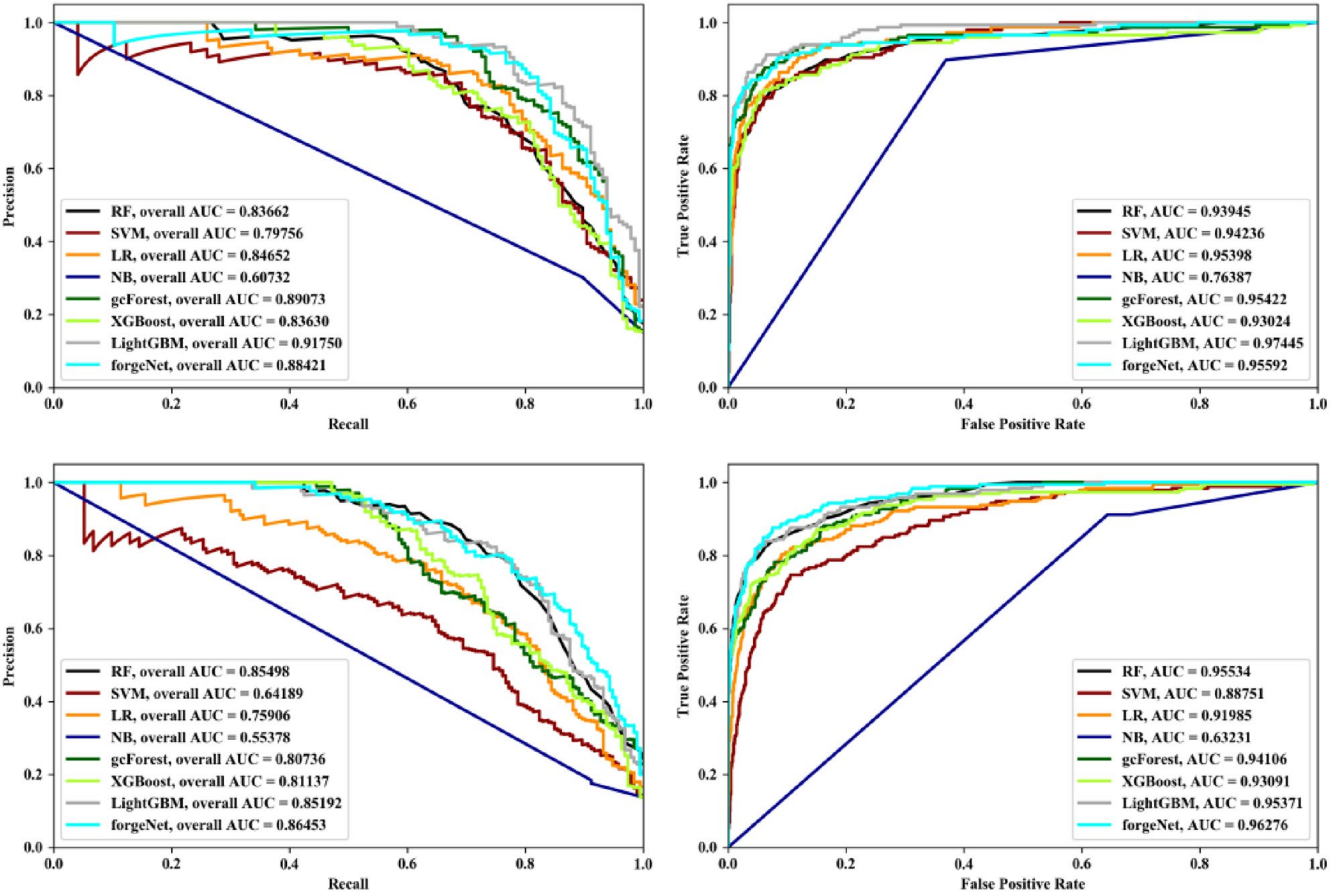
PR curves and ROC curves of eight methods with Data1 and Data2 by threefold cross validation method.

**Figure 5:**
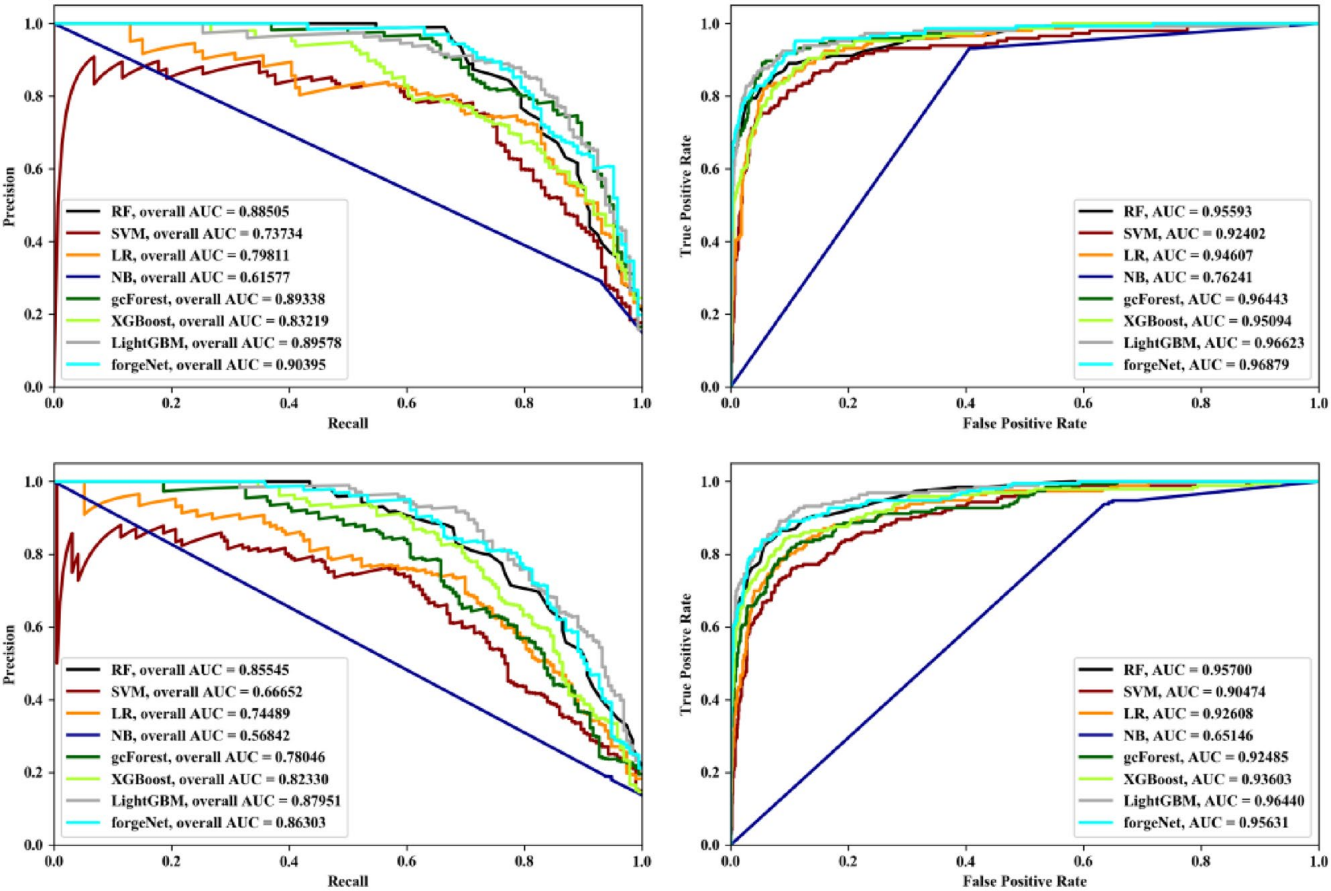
PR curves and ROC curves of eight methods with Data1 and Data2 by threefold cross validation method.

**Figure 6:**
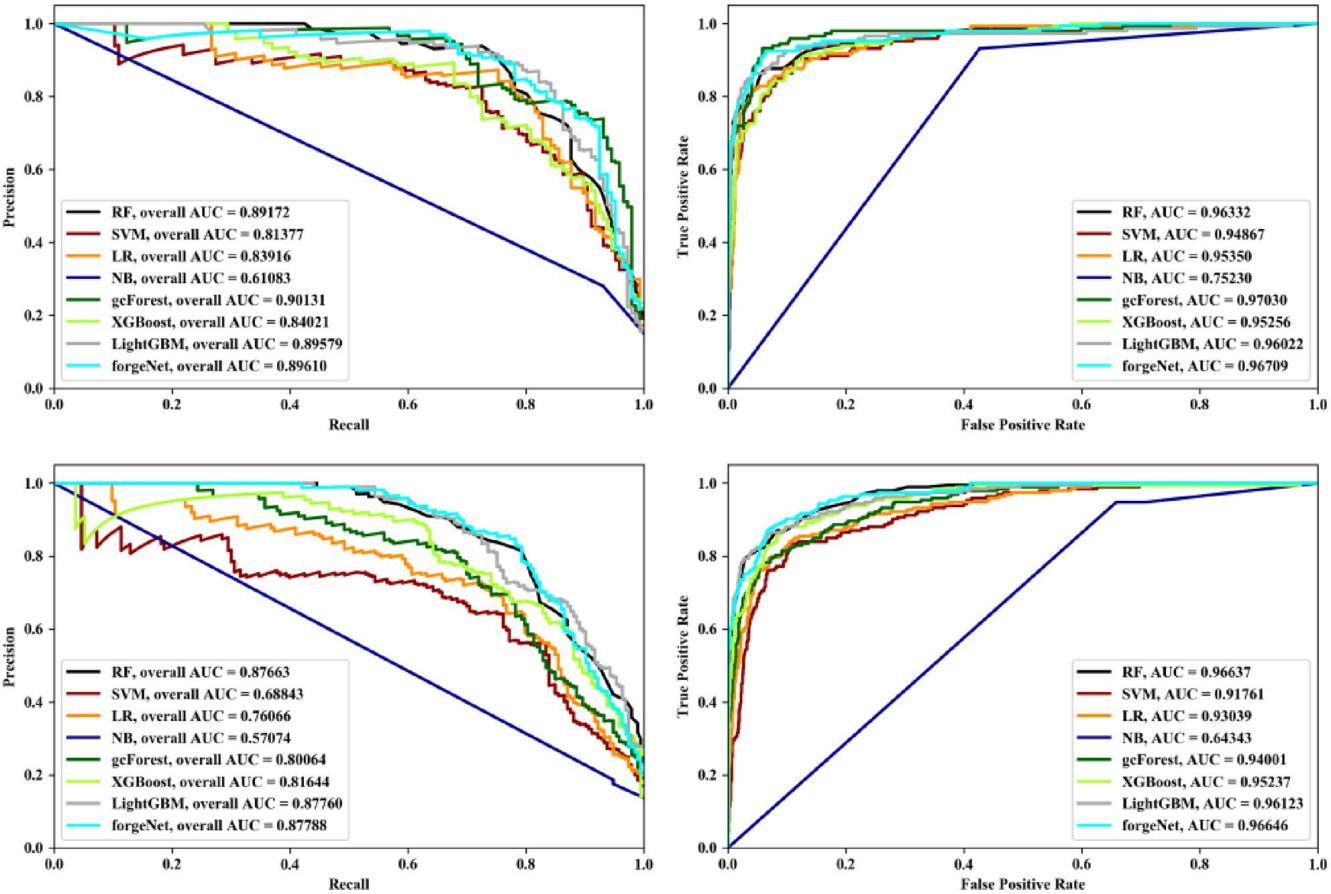
PR curves and ROC curves of eight methods with Data1 and Data2 by threefold cross validation method.

Receiver operating characteristic (ROC) and Precision-Recall (PR) curves are two important curves to evaluate the performance of machine learning algorithm. ROC curve is based on false positive rate (FPR) and true positives rate (TPR). PR curve is based on Recall and Precision. Area under curve (AUC) is defined as the area under the ROC curve or PR curve surrounded by the coordinate axis. PR and ROC curves of eight methods with Data1 and Data2 by threefold cross validation, fivefold and tenfold cross validation methods are depicted in Figs. 4, 5 and 6, respectively. From Fig. 4, it could be seen that LightGBM performs best with Data1 in terms of PR and ROC curves. forgeNet could obtain the second better performances. With Data2, forgeNet could obtain the best ROC and PR curves. From Fig. 5, with Data1, gcForest, LightGBM and forgeNet have the similar PR and ROC curves. From AUC values, it could be seen that forgeNet performs better than gcForest and LightGBM. With Data2, LightGBM and forgeNet have the similar PR and ROC curves. Figure 6 also shows that forgeNet could perform better than other classifiers for compound identification.

### Compound screening for traditional Chinese medicine prescription

Erhuang decoction (EhD) is a traditional heat clearing and detoxifying prescription, which is composed of Radix Scutellariae, Rhizoma Coptidis and licorice. 19 active chemical compounds (Neoglycyrol, Uralenol, Syringic acid 4-β-d-Glucopyranoside, Gancaonin N, Chrysin -6-C-glucoside-8-C-arabinoside, Chrysin-6-C-arabinoside 8-C-glucoside Liquiritin, Baicalin, Isomer of Baicalin, Oroxylin A-7-O-β-d-glucuronide, Chrysin-7-O-glucuronide, Isoliquiritin, Wogonoside, Liquiritigenin, Baicalein, Isoliquiritigenin, Wogonin, Oroxylin A, and Glycyrrhetinic acid) in EhD can dock with ALI related target genes and have high potential biological activity, which have been proved in the reference^39^. Dexamethasone (DXMS) is used as control drug. Molecular descriptors and molecular fingerprints are also utilized to obtain the features of 20 chemical compounds. Data1 and Data2 are utilized as the training sets in order to predict 20 chemical compounds, respectively. SVM, RF and gcForest are selected as comparison methods. The prediction ranks are listed in Table 4. By ranking results, we can see that DXMS ranks last by forgeNet on average, which is consistent with the results of molecular docking in the past research^39^. Thus the results reveal that forgeNet could screen the chemical compounds more accurately than SVM, RF and gcForest. We also analyze the mechanism of action of the highly ranked compounds for treatment of ALI. In the highly ranked compounds, Glycyrrhizin has a protective effect on acute lung injury through the activation and increase of Nrf2 nuclear translocation^45^. Baicalin plays a role in regulating the inflammatory response of ALI by stimulating regulatory T cells and inhibiting the release of IL6 and interleukin-23, which could lead to the decrease of Th17 (T helper cell 17) cells in order to affect the immune balance between Th17 and Treg response^46^. Baicalein can down regulate the mRNA expression of STAT3 and STAT4 in T cell JAK STAT signal pathway in order to promote T cell proliferation, and play an immune and anti-inflammatory role.

**Table 4:** Prediction ranks of 20 chemical compounds by SVM, RF, gcForest and forgeNet.

ID	Chemical compounds	D1	D2	D1	D2	D1	D2	D1	D2
		SVM	SVM	RF	RF	gcForest	gcForest	forgeNet	forgeNet
1	Neoglycyrol	11	13	18	8	12	12	11	8
2	Uralenol	12	15	12	6	16	6	4	7
3	Syringic acid 4-β-d-Glucopyranoside	3	4	7	9	17	11	1	17
4	Gancaonin N	13	16	19	10	15	10	12	6
5	Chrysin -6-C-glucoside-8-C-arabinoside	14	19	15	18	11	19	10	18
6	Chrysin-6-C-arabinoside 8-C-glucoside	15	18	14	19	9	20	9	20
7	Liquiritin	9	6	8	12	4	13	17	13
8	Baicalin	17	9	5	14	8	14	3	11
9	Isomer of Baicalin	18	10	10	15	19	15	5	10
10	Oroxylin A-7-O-β-d-glucuronide	16	17	13	16	10	16	8	14
11	Chrysin-7-O-glucuronide	7	7	9	13	13	9	2	9
12	Isoliquiritin	10	5	16	20	7	17	13	12
13	Wogonoside	20	20	11	17	20	5	6	16
14	Liquiritigenin	19	14	20	11	1	18	19	15
15	Baicalein	2	11	17	7	6	4	14	3
16	Isoliquiritigenin	6	2	1	2	18	1	7	1
17	Wogonin	4	12	3	5	5	7	16	5
18	Oroxylin A	5	8	4	4	2	8	15	4
19	Glycyrrhetinic acid	8	1	6	1	3	2	18	2
20	DXMS	**1**	**3**	**2**	**3**	**14**	**3**	**20**	**19**

Significant values are in bold.

### Performance test of different feature sets

**Figure 7:**
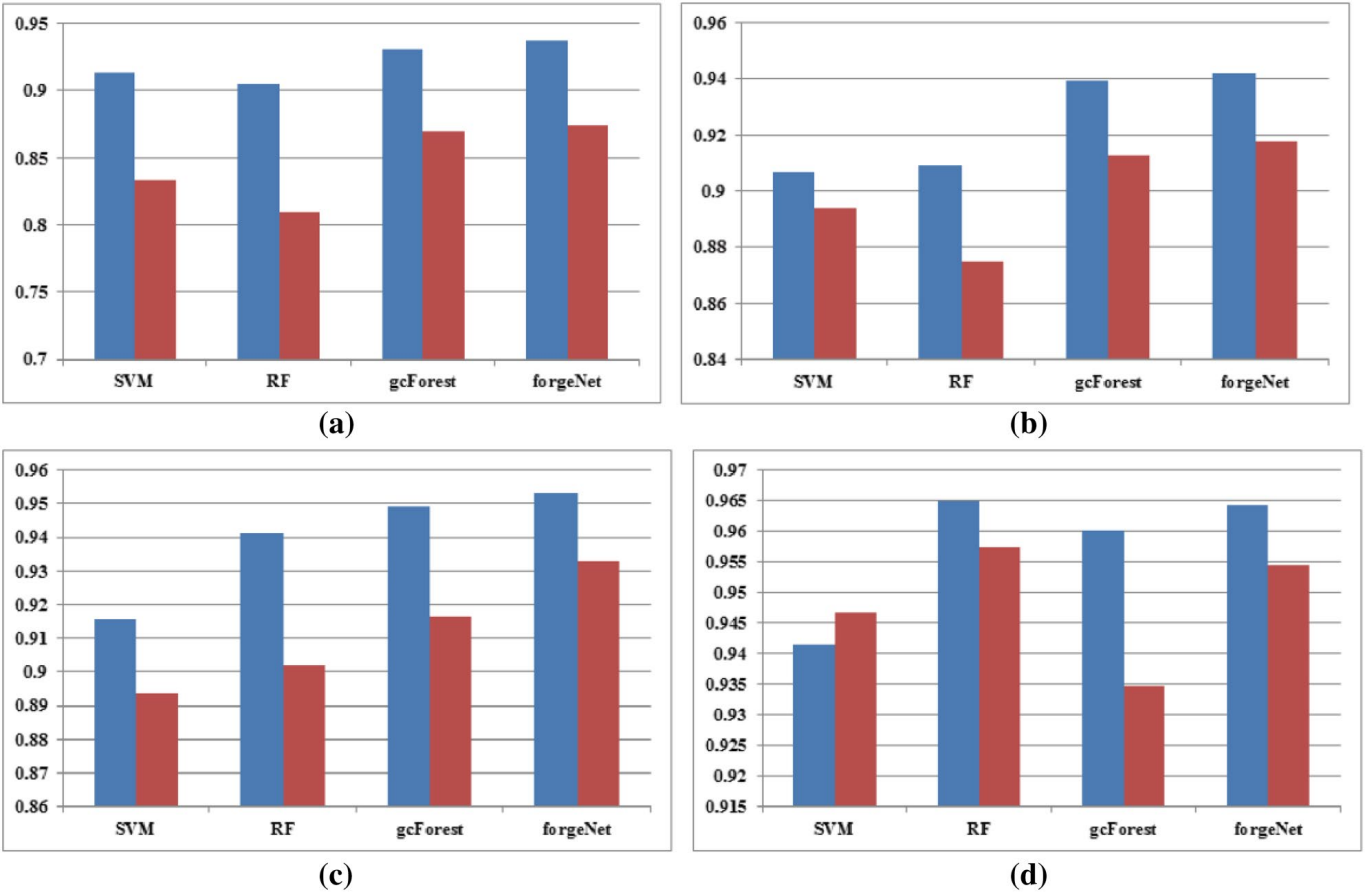
AUC performances of four methods by leave-one-out (**a**), threefold (**b**), fivefold (**c**) and tenfold (**d**) and methods with full feature set (blue) and control feature set (red).

**Figure 8:**
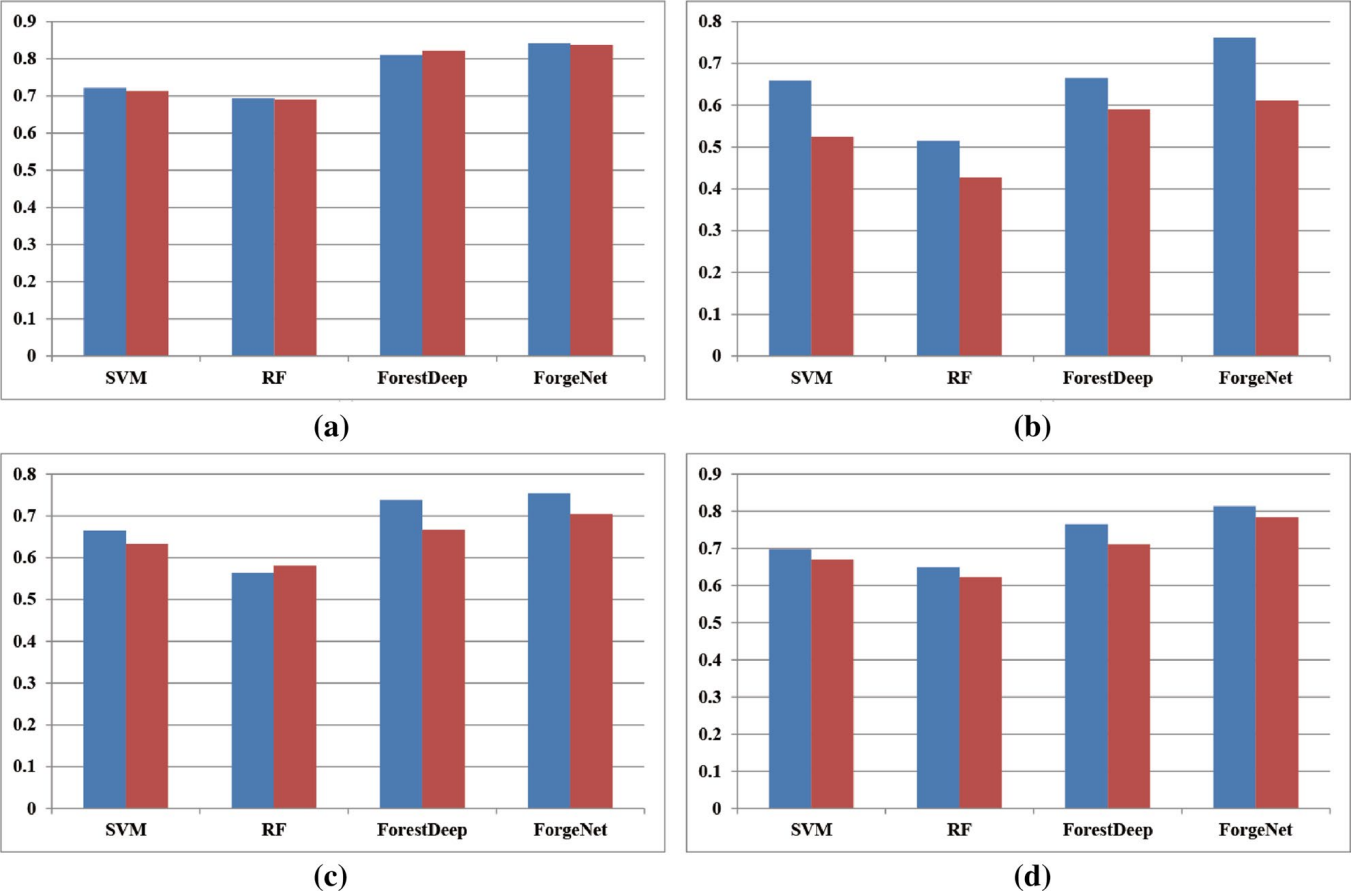
*F*1 performances of four methods by leave-one-out (**a**), threefold (**b**), fivefold (**c**), and tenfold (**d**) methods with full feature set (blue) and control feature set (red).

In order to test the influence of different feature sets on the identification results, we utilized molecular descriptors as control feature set. Molecular descriptors and molecular fingerprints make up full feature set. With these two feature sets, SVM, RF, gcForest and forgeNet are utilized by threefold, fivefold, tenfold and leave-one-out methods. The AUC and F1 results are depicted in Figs. 7 and 8, respectively. From the results, it could be seen that full feature set could improve the compound identification accuracy of methods.

## Conclusions

Network pharmacology has become a frontier and hot spot in the field of traditional Chinese medicine research. This research method can effectively predict the effective components, target and side effects of drugs, and is conducive to the process of modernization of traditional Chinese medicine. In order to construct "disease-gene-target-drug" interaction network more accurately, forest graph embedded deep feed forward network is utilized to infer "disease-compound" network in this paper. According to acute lung injury, two ALI-related target genes (REAL and SATA3) are selected, and the active and inactive compounds of the two corresponding target genes are collected, respectively. Molecular descriptors and molecular fingerprints are utilized to characterize each compound. By threefold, fivefold and tenfold cross validation methods, the experimental results show that forgeNet has the better performance than SVM, RF, LR, NB, XGBoost, LightGBM and gcForest in terms of *SN*, *SP*, *Kappa*, *MCC*, *F*1, AUC, ROC curve and PR curve. ForgeNet is also utilized to identify 19 compounds in Erhuang decoction (EhD) and Dexamethasone (DXMS) and the results reveal that forgeNet could infer the compounds of disease related more accurately. We also test the influence of different feature sets on the identification results and find the feature set based on molecular descriptors and molecular fingerprints could improve the compound identification accuracy of methods.

In the further we will apply the method to prioritize the compounds in other ALI-related and other diseases related TCM prescriptions.

## Data Availability

The data used to support the findings of this study are available from the corresponding author upon request.
